# Temperature Effect on Parasitism in Auricularia Larva of the Sea Cucumber *Isostichopus fuscus*: Implications for Aquaculturing and Management

**DOI:** 10.3390/ani16081133

**Published:** 2026-04-08

**Authors:** Jorge I. Sonnenholzner-Varas, María Panchana, Ricardo Searcy-Bernal

**Affiliations:** 1 Departamento de Acuicultura, Pesca y Recursos Naturales Renovables, Facultad de Acuicultura y Ciencias del Mar, Universidad Técnica de Manabí, Bahía de Caráquez, Manabí 130104, Ecuador; 2 Centro Nacional de Acuicultura e Investigaciones Marinas, Escuela Superior Politécnica del Litoral (ESPOL), 30.5, Vía Perimetral, Guayaquil 90902, Ecuador; mpanchan@espol.edu.ec; 3 Instituto de Investigaciones Oceanológicas, Universidad Autónoma de Baja California, Carretera Tijuana-Ensenada, Ensenada 3917, Mexico; rsearcy@gmail.com

**Keywords:** holothurian, prevalence, intensity, disease severity index, protozoa

## Abstract

A protozoa leads a rapid onset of symptoms, with high mortality rates in the rearing of the planktotrophic larvae of the tropical holothurian *Isostichopus fuscus* for aquaculture. This study aimed to determine the effect of temperature on the infection parameters and the larvae size with/without parasites in its five development stages: T1–EA (250–300 μm), T2–MA-I (300–600 μm), T3–MA-II (600–900 μm), T4–A (1.1–1.3 mm), and T5–LA (≈500 μm). Eight 500 L conical tanks with filtered and UV-sterilized seawater with 0.2 larvae mL^−1^ under two temperature treatments (low: 23.0 ± 2.5 °C, high: 27.0 ± 0.5 °C). Thirty larvae per replicate at intervals of 4–5 days were sampled until day 25. A total of 1200 larvae were examined, and a total of 21,715 parasites were counted. A total of 97% of parasites were found under low temperature conditions. The DSI and survival significantly varied in relation to the temperature and time. The highest prevalence and intensity, 62.5 ± 5.7% and 130.5 ± 13.0, respectively, were observed at T4 at low temperature. At 18 d, larvae without parasites were larger (1494.1 ± 52.2 μm) than larvae with parasites (1237.7 ± 24.4 μm, *p* < 0.05) at 25 d. These findings suggest that elevated temperature functions as a key regulator in mitigating parasitic infections in auricularia larvae of *I. fuscus*.

## Simple Summary

We evaluated the effect of temperature on the disease severity index (DSI), as a global infection parameter derived from prevalence and intensity, as well as survival, and total length of the auricularia larvae of *I. fuscus* with/without parasites across five developmental time points under laboratory conditions. We reared early auricularia larvae of *I. fuscus* in eight 500 L tanks with FSSW at 0.2 larvae mL^−1^ under two temperature conditions (high: 27.0 ± 0.5 °C and low: 23.0 ± 2.5 °C). Larval samples were collected from both treatments across five time points (days 6, 11, 16, 21, and 25). The highest temperature showed the lowest DSI. There were differences in larval length and survival between the two temperature conditions with/without parasites. Elevated temperature does not compromise the viability of *I. fuscus* auricularia larvae and instead identifies 27 ± 0.5 °C as an optimal thermal regime for enhancing larval performance and physiological condition in tropical aquaculture. These findings provide critical insights into one of the main constraints for sustainable *I. fuscus* aquaculture: achieving reliable larval production under the combined pressures of pathogens, disease, and environmental stressors.

## 1. Introduction

Tropical sediment-swallowing holothurians (order Synallactida, family Stichopodidae) play an important ecological role as ecosystem engineers in marine environments [1,2]. They also hold commercial significance, being highly consumed as a traditional delicacy called “Ginseng of the Sea”, which marketed in Southeast Asia as bêche-de-mer in its dried premium form [3,4,5]. Nowadays, they are marketed as a “superfood” for their high quality and concentration of bioactive compounds, which support nutraceuticals and pharmaceutical applications for human and animal health-promoting effects [6,7,8]. Nonetheless, the continued growth of global demand for holothurians, aquaculture has emerged as a necessary strategy, and the industry is challenged to ensure a stable and sustainable supply. 

Based on the principles of the Code of Conduct for Responsible Fisheries, the aquaculture of these holothurians has gained emerging importance (USD $902 million in 2024, with more than 1,800,000 t), with an upward trend, showing solid expansion with a compound annual growth rate of 6.9% from 2025 to reach USD $1780 million in 2033 [9,10]. In fact, this industry is promoting intensive farming methods that are being rapidly developed, but at the same time, it is contending to bottlenecks for its sustainability, such as: (i) the intensification of fishing pressure on natural populations, which has severely depleted many wild stocks due to overfishing, illegal exploitation, and trafficking, affecting more than 70 sea cucumber species and jeopardizing the long-term availability of broodstock [11,12]; (ii) the aquaculture of sea cucumbers using hatchery-produced seeds, which could both enhance the declining wild populations and provide sufficient bêche-de-mer product to satisfy increasing market demand, but this remains technically challenging, particularly due to the limited development of scalable larval rearing technologies and the lack of established reliable hatchery protocols for several species, particularly regarding to environmental conditions, e.g., temperature [13,14,15,16,17]; and (iii) climate change and extreme ocean–atmosphere events that are altering coastal marine systems, creating highly variable conditions in temperature, dissolved oxygen, and water quality, which can negatively impact the successful production of healthy larvae and seeds in hatchery operations [17]. Therefore, the global sea cucumber aquaculture industry is not progressing adequately to meet the high consumer demand.

High larval stocking densities in sea cucumber hatcheries can increase the risk of disease outbreaks by facilitating pathogen proliferation and transmission under intensive culture conditions [18]. Therefore, optimizing stocking densities is essential to maintain production efficiency while minimizing physiological stress and infection risk. Larval densities should be adjusted according to interspecific differences in auricularia size and biological traits. For example, densities of up to 0.60 larvae mL^−1^ have been reported for species such as *Holothuria leucospilota, H. nobilis,* and *H. scabra,* whereas species with larger larvae, such as *Apostichopus japonicus* and *Isostichopus fuscus* generally require lower densities ranging from 0.1 to 0.2 larvae mL^−1^ [19,20,21,22,23,24,25,26].

However, even under recommended stocking densities for holothurian larvae, environmental factors—particularly temperature—play a critical role (as abiotic stressor) in determining larval performance (as host) and disease susceptibility [27]. The edible tropical holothurian, *I. fuscus* (Ludwig, 1875), has been reared successfully under controlled temperature conditions, but larval disease caused by parasitic infection may occur at low densities [26,27]. The literature reports that elevated temperatures may extend the duration of parasite transmission, thereby increasing disease prevalence, although this is not always consistent [17]. This means that responses to temperature are not universal across biological systems. In some cases, rising temperatures have been associated with reduced prevalence of certain diseases, suggesting that the interaction between temperature and parasitism is complex and context-dependent [27,28]. However, this interaction remains poorly understood and requires further experimental investigation. Ref. [29] demonstrated that the survival rates, population density, and growth of the parasitic protozoan *Mesanophrys* sp. infecting the crab *Portunus trituberculatus* were higher at low temperatures (≈12 °C) and progressively decreased as the temperature increased between 16 °C and 26 °C. Additionally, the authors of [30] determined the effect of temperature on trematodes *Echinoparyphium aconiatum* in its snail host under high temperature. They discovered that the parasites exhibit optimal growth or virulence at low temperatures, while at high temperatures, their infectious success is lower, which benefits the host. Therefore, high temperatures can modify host–parasite dynamics, but their effects on the prevalence or intensity of infection will depend on the host’s physiology and resistance.

In the eastern tropical Pacific, including Ecuador, *I. fuscus* is the only commercially overexploited sea cucumber species [31,32], and it is the second most expensive on the market, just after *A. japonicus* (as high as $2400 per kg [33]). Despite this status, its demand has continued to grow in Asian markets for its nutritional quality and bioactive compounds for human health-promoting effects [34,35,36]. Accordingly, its aquaculture has become an area of increasing interest, and it is currently progressing in Ecuador, where the species has demonstrated strong feasibility and potential as a high-value candidate for the emerging blue economy seafood industry [15,37]. In 1999, Ecuador explored its culture to diversify aquaculture production since private initiative [26,38,39], but this attempt was hampered by massive larval mortality due to diseases in the hatcheries [38,39]. Fifteen years later, the Government of Ecuador, through the Secretariat of Aquaculture, promoted studies to establish reliable aquaculture protocols for *I. fuscus*. The efforts were focused on addressing key challenges during auricularia larval rearing: (i) stocking density, (ii) nutrition, and (iii) disease caused by parasitism.

*I. fuscus* is an indirect-developing holothurian with a feeding dipleurula-type larva (auricularia) characterized as an oligotrophic, transparent pelagic stage [40]. This early planktonic stage is extremely sensitive to low temperatures in the equatorial Pacific (<21 °C) where the larva’s metabolic activity is significantly reduced producing a dormancy condition that could last several months [41]. This has significant operational implications for aquaculture, because in hatcheries, it delays larval development, prolongs culture cycles, and increases the risk of mortality through infections [42]. Therefore, in *I. fuscus* larval rearing, temperatures are generally maintained at around 24–26 °C, where the development is faster and more stable [43]. Nevertheless, this larva harbors an early-life core microbiome that includes free-living undetermined bacteria and amoeboid protozoa, which play important roles in host development, health, and environmental adaptation [44]. The larval core microbiome in holothurians of the order of Synallactida include four dominant bacterial taxa: Alteromonadales, Rhodobacterales, Oceanospirillales, and Flavobacteriales [45]; but so far, there are no studies with molecular sequencing of the parasitic organism that is found in the auricularia larva of *I. fuscus* to allow for more precise taxonomy confirmation. Nonetheless, those unicellular protozoan evidence a true and direct parasitism (not endosymbionts) which invades, penetrates, and causes severe damage to the larval digestive tract tissue (intestinal shrinkage, atrophy, and organ collapse), causing pathological effects and mortality and probably acting as opportunistic and facultative parasites, exploiting environmental conditions and host stress [27,42]. Two stages as vacuolar structures were observed (approximately 10 μm in diameter) surrounding the digestive organs of the larvae, appearing as: (i) a mobile form with appendages (flagella or filopodia) and (ii) a slow-moving amoeboid form [42]. In aquaculture, the free-living stage of these ameboid parasites’ spreads through the water column, and it can enter hosts through wounds or natural orifices or through accidental ingestion, subsequently attacking the digestive system [18,46]. 

The thermal physiological and immunological resilience of the auricularia larva of *I. fuscus* is a result of its metabolic plasticity, efficient physiological regulation, and functional innate immunity, which allows it to tolerate environmental variability and limit opportunistic infections [47] where the culture conditions do not exceed its adaptive limits in low temperatures [48]. This allows them to tolerate temperature fluctuations without immediate physiological collapse, reducing the impact of thermal stress as a predisposing factor for disease [49]. Nonetheless, high parasite infection can alter energy allocation, impair development, reduce growth rates and swimming performance, and decrease overall fitness. Such effects may lead to elevated mortality when temperatures fluctuate below optimal conditions [50,51,52,53]. 

Resolving host–parasite interactions during larval development is critical for improving growth, immune competence, and disease resistance in this sea cucumber species. However, these processes are governed by complex and poorly understood interactions that require targeted experimental approaches. Here, we address this gap by experimentally assessing the effects of temperature on parasite abundance in planktotrophic larvae of *I. fuscus*. Based on previous studies [29,30,42,54,55,56], a conceptual model on thermal tolerance characterized by a close ecological and physiological coupling is proposed, where the bacteria–protozoan association represents a functionally integrated consortium that could enhance parasite survival under adverse thermal conditions for the host and facilitate transmission or die when the thermal conditions no longer favor them. Deciphering these processes in auricularia larvae of *I. fuscus* is critical for improving growth, immune resilience, and disease resistance. Light refuges, together with the phycobiome and holobiome, likely represent key, non-incidental drivers of host–microbe–parasite interactions that shape disease dynamics. This conceptual model provides a basis for developing ecosystem-based strategies to optimize larval performance in hatchery systems.

The objective of this study was to evaluate the effect of temperature on the disease severity index (as a global infection parameter derived from prevalence and intensity), survival, and total length of auricularia larvae of *I. fuscus* with and without parasites at five developmental phases under laboratory conditions, with an emphasis on understanding the disease dynamic caused by a protozoan species. The findings provide insights into one of the major challenges for sustainable *I. fuscus* aquaculture: ensuring larval production in the face of pathogens, diseases, and environmental stressors.

## 2. Materials and Methods

### 2.1. Sea Cucumber Collecting

Auricularia-stage larvae of *Isostichopus fuscus* were obtained from a broodstock of dark brown morphotype (N = 100), with an average contracted body length of 20.1 ± 1.5 cm and wet weight of 356.3 ± 20.4 g. The specimens were collected by hookah diving at depths ranging from 12 to 25 m, 5 days before the new moon, based on the lunar calendar [43] from El Pelado islet, Santa Elena, Ecuador (01°55′ S, 80°47′ W), in August 2016. The animals were transported to the aquaculture facility at the Centro Nacional de Acuicultura e Investigaciones Marinas—Escuela Superior Politécnica del Litoral (CENAIM-ESPOL), Santa Elena, Ecuador.

### 2.2. Spawning and Larval Rearing Conditions

Fifteen holothurians were placed in 500 L high-density square polystyrene dark tanks at a stocking density of 323 g wet weight m^−2^ to prevent polyspermy [57,58]. The tanks were filled with filtered, UV-sterilized seawater (FSSW) at ambient temperature under a natural photoperiod and light aeration. The animals were not handled during spawning to avoid the interruption of gamete release. Because larvae reared at high densities are often more susceptible to disease and parasitism, preliminary 10-day trial was conducted to determine the optimal stocking density. Four densities were tested: 0.2, 0.5, 1.0, and 1.5 larvae mL^−1^. The larvae were fed a mixed algal diet at <5 × 10^4^ cells mL^−1^ at 23.0 °C. The results indicated that densities of >0.5 larvae mL^−1^ failed to develop properly, remained small, and showed high levels of malformations and parasitism, with zero survival. For the main experiment, see the experimental design. The upwelling system prevented larval settlement at the tank bottom and prolonged suspension of microalgae in the water column under gentle aeration. The moribund larvae that sank, along with the dead larvae and fecal material, were siphoned from the bottom. Seawater was renewed three times per week: twice with a partial exchange (50%) and once with a full exchange (100%), using a 100–250 μm mesh sieve. The larvae were fed daily, twice a day (08:00 and 15:00), with rations adjusted based on stomach fullness. The diet consisted of a microalgal mixture (4:1:1) of *Chaetoceros gracilis*, *Rhodomonas* sp., and *Tisochrysis lutea* (=*Isochrysis galbana*) at concentrations ranging from 1 × 10^4^ to 4 × 10^4^ cells mL^−1^. Water quality was monitored using a multiparameter water quality tester (model HI 9828). Average conditions during the experiment were the following: salinity 34‰, pH 8.2, dissolved oxygen 5–6 mg O_2_ L^−1^, and irradiance 0.5 μE m^−2^ s^−1^, under a natural 12 h light:12 h dark cycle.

### 2.3. Conceptual Model: Thermal/Light Regimes in Hatchery Conditions for Auricularia Larvae

Our conceptual model based on [28] simulates two thermal scenarios to assess the influence of temperature on parasitism in *I. fuscus* auricularia larvae under controlled hatchery conditions. Both treatments were conducted under reduced light levels to mimic the attenuated illumination that regulates larval vertical positioning in the water column. At higher temperatures (27 °C), larval development is accelerated, leading to shorter developmental periods, higher survival rates, and reduced transmission of protozoan parasites, resulting in lower parasite prevalence despite increased metabolic demand. In contrast, at lower temperatures (21 °C), larval development is prolonged, creating conditions that favor the persistence and transmission efficiency of protozoan parasites, thereby increasing the likelihood of infection. Larval behavior was incorporated into the model by considering vertical positioning within large cylindrical tanks, allowing individuals to redistribute in response to thermal and light gradients. This behavioral response reflects natural depth selection strategies that help balance energy acquisition with the risk of infection. Overall, the model predicts that parasite prevalence in auricularia larvae decreases under hatchery conditions characterized by higher temperatures and reduced light levels, highlighting a mechanistic interaction among temperature, light exposure, larval behavior, and protozoan dynamics.

### 2.4. Experimental Design

A completely randomized experimental design was implemented using early auricularia larvae of *I. fuscus* reared in eight 500 L dark conical smooth truncated cylinder tanks with FSSW at a stocking density of 0.2 larvae mL^−1^. To evaluate the effects of temperature stress on parasite infection dynamics in auricularia, two experimental treatments were applied. In treatment #1 (high-temperature), four tanks were equipped with heat-resistant hoses (Chun system) connected to a boiler to maintain the water temperature at 27.0 ± 0.5 °C and avoid daily temperature fluctuations. In treatment #2 (low-temperature), four tanks were maintained under ambient temperature conditions (23.0 ± 2.5 °C). Each treatment included four replicates (n = 4 tanks). The thermal conditions applied in this experimental study were realistic, and they were implemented under a thermal coefficient of 1.286 between both treatments. As temperatures in the ambient treatment dropped to 21 °C for more than 9 days in August 2016, the larval samples were collected from both treatments at five time points (days: 6, 11, 16, 21, and 25) after the onset of the experiment for measurement and survival assessment. From each tank and at each time point, 30 larvae were randomly obtained (total 600 larvae per treatment, 30 × 4 = 120 per time point). All larvae were differentiated between larvae with and without parasites for each treatment (abundance, prevalence, intensity, and the disease index). Of those 120 larvae, 80 were randomly obtained per treatment, 10 larvae per tank (40 with and 40 without parasites) for the measurement of the total larval length.

### 2.5. Larval Sampling and Measurement

**Figure 1:**
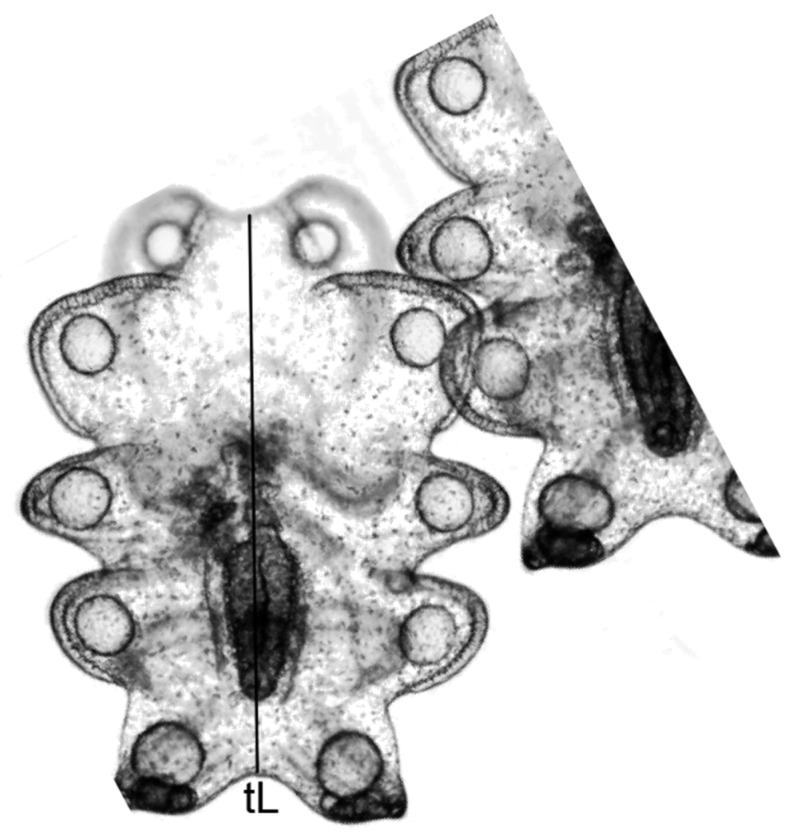
Total length (tL) measurement of a mature auricularia larva, A—T4, of the sea cucumber *Isostichopus fuscus* (Ludwig, 1875) at five different time points during the trial that lasted for 25 days.

The planktotrophic larva of *I. fuscus* was measured in total length, tL (Figure 1).

Five developmental stages were identified based on larval size and morphological criteria: (T1: 6 days) early auricularia (EA): 250–300 μm; (T2: 11 days) middle auricularia I (MA I): 300–600 μm; (T3: 16 days) middle auricularia II (MA II): 600–1000 μm; (T4: 21 days) mature auricularia (A): 1000–1300 μm; and (T5: 25 days) late auricularia (LA): ~500 μm. SMA I, MA II, and A stages were defined by the extension of the left somatocoel to less than half the length of the gut, without an increase in axohydrocoel length, and the presence of 10–12 hyaline spheres. The LA stage was characterized by the extension of the left somatocoel beyond half the gut length, accompanied by the elongation of the axohydrocoel from its original spherical shape and the beginning of its retraction. For each sampling event, three 1 mL aliquots containing 10 larvae each were examined from every tank (four replicates per treatment), yielding a total of 30 larvae per tank and 120 larvae per treatment across the five sampling dates. Larval survival was estimated based on the number of dead individuals recorded from each set of aliquots (n = 3 per tank). Larval length was measured with an eyepiece micrometer under a compound microscope, and parasites were counted using an optical microscope. The images were captured with a high-resolution digital camera (MDX503, Lanoptik Technologies Ltd., Guangzhou, China) mounted on a trinocular microscope (CX31RTSF, Olympus Corporation, Tokyo, Japan), and they were processed using the Nahwoo iWorks 2.0 software for data acquisition and analysis.

### 2.6. Infection Parameters

**Figure 2:**
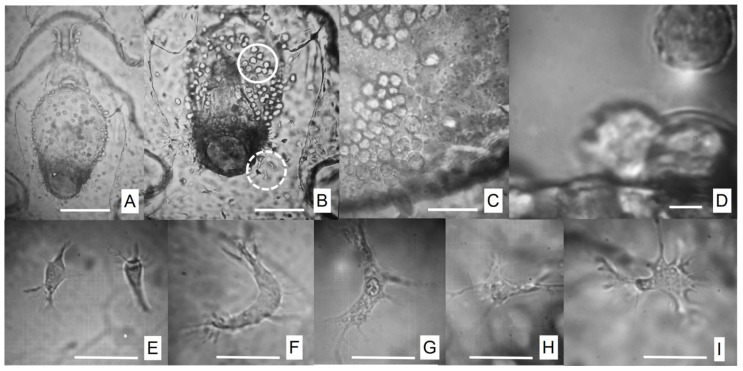
The view of two protozoan stages in diseased auricularia larva of the sea cucumber *Isostichopus fuscus*. (**A**–**D**) Spherical slow-moving ameboid shape in the stomach and intestine ((**B**), white full circle). (**E**–**I**) Mobile shape with flagelae or filipodiae ((**B**), white cutline circle), with a cytoplasm highly vacuolated and a circular-oval nucleus present in the entire larva. Scale bars: (**A**,**B**) 200 μm; (**C**,**D**) 20 μm; and (**E**–**I**) cells (10 μm).

Figure 2 shows the protozoans observed in diseased auricularia larvae of *I. fuscus*. Our conceptual model proposes that large, mobile larvae reared in tanks as gregarious populations are more strongly associated with parasite transmission dynamics than smaller hosts. In this context, microparasite transmission may occur primarily through direct contact. Mobile parasites can move freely and actively seek new hosts, often through swimming [46]. In this study, infection parameters (prevalence, mean intensity, and the disease severity index) were calculated at each of the five sampling time points using the following formulas:

Mean Abundance = T_NP_/N,

where T_NP_ is the total number of parasites, and N is the total number of hosts screened.

Prevalence (P) = (N_IL_/n) × 100,

where N_IL_ is the number of infected larvae, and n is the total number of hosts screened with parasites, expressed in percentage.

Mean Intensity (MI) = T_NP_/n,

where T_NP_ is the total number of parasites, and n is the total number of hosts with parasites screened.

Disease Severity Index (I_DS_) = P × MI,

where P is the prevalence, and MI is the mean intensity. This composite variable allows for a more complete view of a disease’s impact on a population, considering not only how many larvae present with the disease, but also how severe the condition is. The severity can vary over time. If the disease prevalence is 20%, and the average intensity is 5, then I_DS_ is low (level I). See Table 1.

### 2.7. Statistical Analysis

A repeated-measures analysis of variance (ANOVA) was performed to evaluate the effects of the infection parameters (prevalence, intensity, and the disease severity index) across five sampling time points, with two fixed factors: temperature and parasite presence. Prior to the analysis, the assumptions of normality and homoscedasticity were tested at α = 0.05 using the Kolmogorov–Smirnov and Levene’s tests, respectively. When these assumptions were violated (*p* < 0.05), the data were arcsine-transformed before the analysis. Mauchly’s test of sphericity was non-significant (*p* > 0.05). In cases where the assumption of sphericity was not met, the degrees of freedom were adjusted using the Greenhouse–Geisser correction. Post hoc comparisons of mean response variables were conducted using Tukey’s HSD test. The data are presented as mean ± standard error. All statistical analyses were carried out using Statistica v.10 (StatSoft Inc., Tulsa, OK, USA) and IBM SPSS Statistics v.19 (IBM Corp., Armonk, NY, USA).

### 2.8. Ethics Statement

All experimental studies were conducted in accordance with the recommendations of the Guide for the Care and Use of Laboratory Animals of CENAIM–ESPOL. The protocols were reviewed and approved by the Subsecretary of Aquaculture of the Government of Ecuador (Approval No. CUP #133600000.864.423; Resolution No. 2012078, 3 December 2012).

## 3. Results

### 3.1. Etiology and Clinical Signs

**Figure 3:**
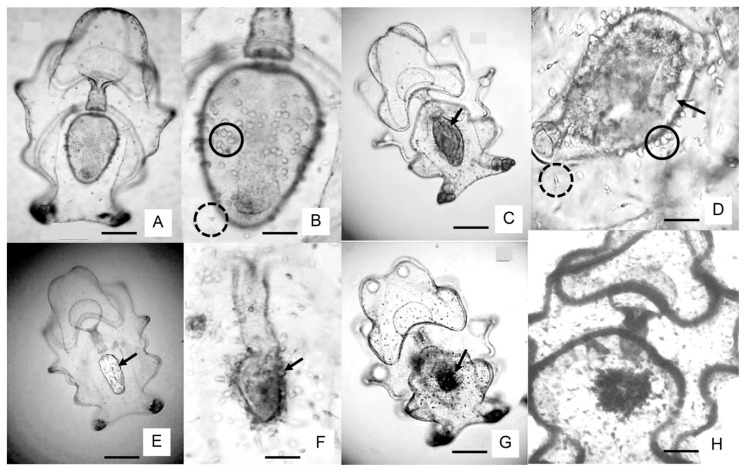
Micrographs of the prevalent disease complex on auricularia larvae of the sea cucumber *Isostichopus* fuscus at the low temperature regime. (**A**,**B**) Stomach ulceration symptoms, SUSs, in MA I—T2. Black arrow shows initial condition, anorexia/loss of appetite, empty digestive tract, motor disturbance, and erratic swimming; rounded stomach invaded by amoeboid protozoa, cluster-like aggregates of 15–80 parasites in the equatorial region (passive form in dark circle line and active form in circle clipped line), and larva reduced size; (**C**–**E**) Rotting-edge syndrome, RES, in MA I—T2 and MA II—T3, close-up view of the inflammatory process thickening of stomach wall with change in coloration to brown, delayed formation of larval structures (black arrow); (**F**–**H**) stomach atrophy syndrome, SAS in MA I—T1 and MA II—T2, stomach shrinkage, collapse/implosion, larval decay, high mortality, and autolysis with thickened/darkened edges and inflammatory cells in the epithelial tissues become ulcerated with epithelial cell necrosis in a severely larva infected. (**H**) Dark purple edges when stained with hematoxylin and eosin.

**Figure 4:**
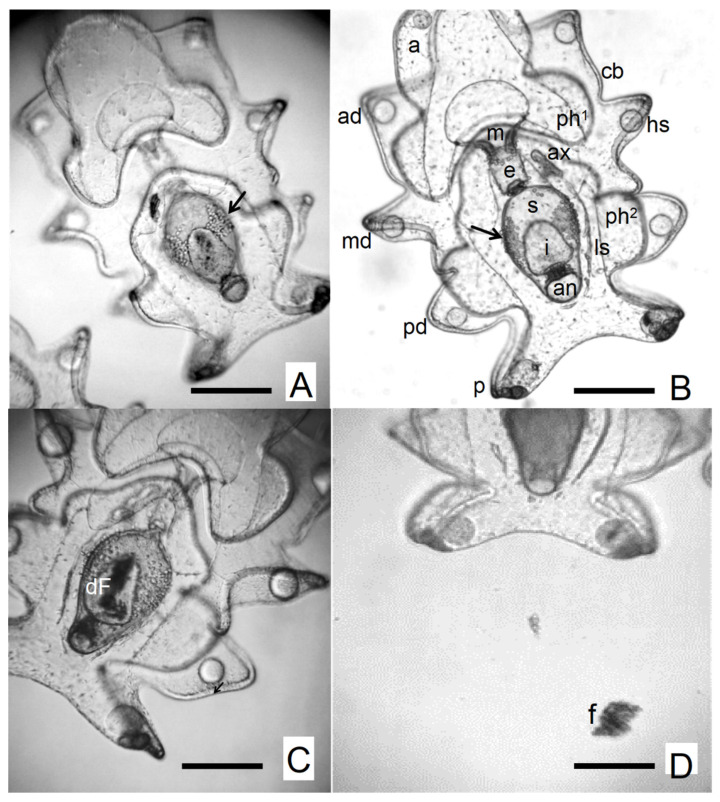
Prevalent disease of stomach ulceration symptoms (SUSs) in (**A**) middle auricularia II, MA II—T3, and (**B**) mature auricularia, A—T4 of the sea cucumber *Isostichopus fuscus* at high temperature regime. (**C**,**D**) Intestine with digested food; dF and feces, f. Arrows show that stomachs are invaded by amoeboid parasites. Letters indicate: cb = cilary band; m = mouth; e = esophageus; s = stomach; i = intestine; an = anus; ax = axohydrocoel; ls = left somatocoel; hs = hyaline spherules; ph1 = pre-oral hood; ph2 = post-oral hood; ad = anteriodorsal fold; a = anterior fold; md = mid-dorsal fold; pd = posteriodorsal fold; p = posterior fold. Scale bar = 100 μm.

Optical microscopy revealed infected larvae exhibiting two forms of protozoan parasites (Figure 2A,B): (i) small, opaque, rounded slow-motile ameboid form (with pseudopodia; Figure 2C,D) and (ii) an irregular motile form (with filopodia; Figure 2E–I), measuring 10–12 μm in diameter. The occurrence of the disease happened in two waves influenced by the experimental temperature (Table 2). The first peak corresponded to a high invasion of slow-motile parasites in the body wall (Figure 2A–C and Figure 3A,B) and large clusters of irregular motile parasites with filopodia in the digestive tract (mouth and esophagus; Figure 2A,B and Figure 3A,B) with severe effects (scales III–V; see Table 2) in the stages EA and MA I at low temperature conditions (Figure 2A–H; Table 2). By 5 days post-hatch, all infected larvae exhibited lethargy and erratic swimming, with reduced feeding (Figure 3A,B). Three clinical signs were observed in the stomach: (1) stomach ulceration (Figure 3A,B); (2) stomach atrophy (Figure 3C–E), and (3) stomach rotting edge (Figure 3F–H). The stomach ulcer progressed from an active, rounded and clear pear-like stomach to a vague structure with darkened, rough, thickened borders and a shrunken lumen (Figure 3H). By 7 days post-hatch, the larvae showed empty, shrunken stomachs. In severe cases under starvation, the body edge thickened and darkened, and the stomach epithelium became hyperplastic and eventually disintegrated by autolysis (Figure 3F–H). The second peak, observed at higher temperatures, displayed low to moderate effects (scales I–II; see Table 2), with slow-motile protozoans localized in the equatorial region of the stomach and intestine in the MA II and A stages (Figure 4A–D). All larvae remained actively feeding and produced substantial feces (Figure 4D).

### 3.2. Infection Parameters

Parasite Abundance. A total of 1200 auricularia larvae of *I. fuscus* were examined in this trial. The highest parasite abundance was observed in the low-temperature treatment, with the peak occurring at T3 (1768.8 ± 342.7, Table 2). The lowest parasite counts were recorded in high-temperature conditions at T5 (10.3 ± 4.2, Table 2).

Prevalence. On average, the highest prevalence of parasites was recorded in low-temperature conditions (0.79 ± 0.11; F_(1,6)_ = 10.318, *p* = 0.01832; Table 3), particularly at three stages: T2 (0.70 ± 0.13, Table 2), T3 (0.90 ± 0.11, Table 2), and T4 (0.90 ± 0.12, Table 2) (F_(4,24)_ = 3.3576, *p* = 0.02562; Table 3). A significant interaction was detected with temperature. In low temperature conditions, the highest prevalence occurred at T4 (0.94 ± 0.034, Table 3), whereas the lowest prevalence was recorded at T2 (0.68 ± 0.14, Table 3). In high temperature conditions, prevalence peaked at T2 (0.90 ± 0.05, Table 3) and reached its minimum at T5 (0.20 ± 0.07, Table 3) (F_(4,24)_ = 5.7312, *p* = 0.00220; Table 3).

Media Intensity. On average, the highest disease intensity was recorded in low-temperature conditions (43.30 ± 10.60; F_(1,6)_ = 104.44, *p* = 0.00005; Table 3), particularly at stages T2 (33.60 ± 7.90, Table 2), T3 (71.40 ± 14.40, Table 2), and T4 (60.70 ± 12.90, Table 2) (F_(4,24)_ = 3.9644, *p* = 0.1312; Table 3). A significant interaction was detected with temperature. In low temperature conditions, the highest intensity occurred at T3 (71.40 ± 12.70, Table 3), while the lowest intensity was recorded at T1 (11.10 ± 4.00, Table 3). In high temperature conditions, intensity reached its maximum at T5 (6.30 ± 5.25, Table 3) and minimum at T1 (1.30 ± 0.14, Table 3) (F_(4,24)_ = 3.8785, *p* = 0.1440; Table 3).

The Disease Severity Index. On average, the highest I_DS_ value was recorded in low-temperature conditions (35.07 ± 8.91; F_(1,6)_ = 60.813, *p* = 0.00023; Figure 4, Table 3), particularly at T3 (59.00 ± 11.42, Table 2) and T4 (57.00 ± 12.57, Table 2) (F_(4,24)_ = 5.0002, *p* = 0.0448; Figure 4, Table 3). A significant interaction was observed, with I_DS_ strongly shaped by temperature. At low temperature, the maximum value occurred at T3 (59.00 ± 11.42, Table 3), while the minimum was recorded at T1 (7.00 ± 2.71, Table 3). In high temperature conditions, I_DS_ peaked at T2 (2.13 ± 0.26) and reached its lowest value at T5 (0.30 ± 0.14, Table 2) (F_(4,24)_ = 4.7879, *p* = 0.0554; Figure 4, Table 3).

Larval Size. A delayed larval organogenesis (buccal cavity, mouth, esophagus, stomach, intestine, and cloaca) and a slowed down growth was observed in low-temperature conditions. The development of infected larvae, particularly between T1 and T2, was delayed compared with parasite-free larvae in high temperature conditions, which exhibited faster growth and fully developed structures, including left somatocoel extension to approximately half the stomach length, axohydrocoel elongation, and the presence of 10 large hyaline spherules (Figure 3). In high-temperature treatment, larval development and growth were continued non-retarded (Table 2). The total length of auricularia larvae was significantly affected by both the parasite presence and temperature (Figure 4). Larger larvae were consistently observed in the absence of parasites (F_(1,12)_ = 17.904, *p* = 0.00117; Figure 4, Table 3) under high-temperature conditions (F_(1,12)_ = 11.463, *p* = 0.00541; Figure 4, Table 3). The maximum total length was reached at stage T4 (MA), with mean lengths of 1064.8 ± 11.7 μm at low temperature and 1165.5 ± 11.8 μm at high temperature (F_(4,48)_ = 1565.8, *p* < 0.0001; Figure 4, Table 2 and Table 3). Parasite-free larvae completed development in 18 days, whereas parasitized larvae required 25 days. No significant interaction was found between temperature, parasite presence, and time (F_(4,48)_ = 1.5690, *p* = 0.19775; Figure 4, Table 3).

**Figure 5:**
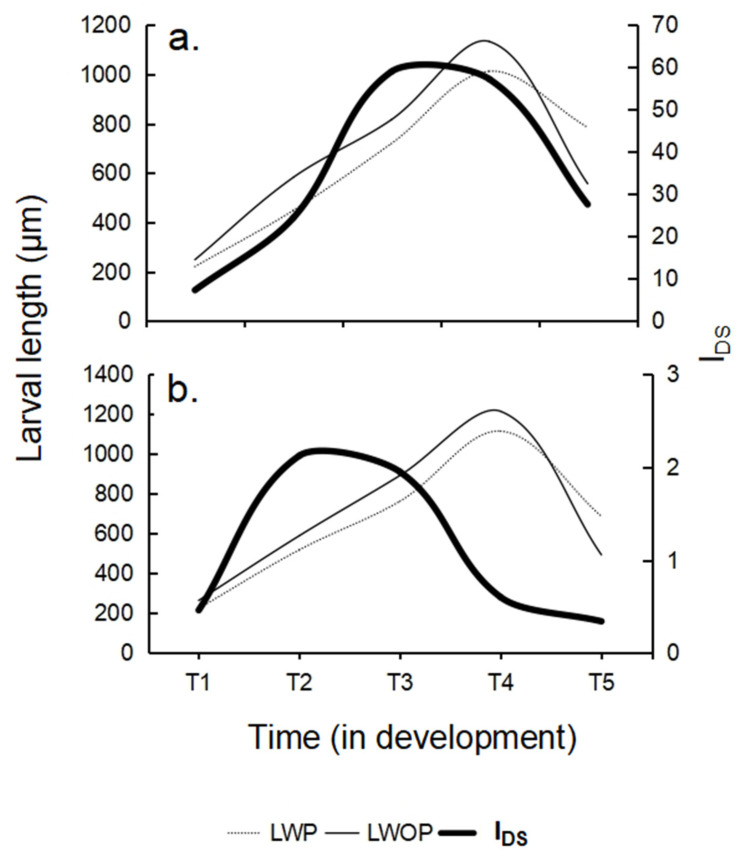
The relationship between larval size and I_DS_ in auricularia of the sea cucumber *Isostichopus fuscus* with (LWP) and without parasites (LWOP) at five different phases of development and under two temperature regimes: (**a**) low temperature and (**b**) high temperature.

*Survival.* High mortality was reported at each sampling time before larvae reached the next stage, which was significantly influenced by temperature (F_(1,6)_ = 773.73, *p* < 0.0001; Figure 5, Table 3). The lowest survival occurred under low-temperature conditions, reaching 14.8 ± 1.9% at T5, whereas the highest survival was recorded under high-temperature conditions, reaching 79.5 ± 1.3% at T5 (F_(4,24)_ = 240.94, *p* < 0.0001; Figure 5, Table 3). A significant interaction was also detected between temperature and the auricularia developmental stage (T1–EA to T5–LA) (F_(4,24)_ = 73.600, *p* < 0.0001; Figure 5, Table 3).

## 4. Discussion

### 4.1. Thermal and Light Refuges as ‘Window of Opportunities’

Adult *I. fuscus* inhabiting equatorial region are predominantly nocturnal [43]. They display a predictable monthly spawning pattern that persists year-round; however, only a fraction of the population spawns during each event, thereby sustaining recruitment over time [59]. This reproductive activity occurs within a defined “window of opportunities” that is strongly modulated by environmental cues, particularly light intensity, photoperiod, spectral composition, and temperature. For this reason, their long-lived oligo- planktotrophic pelagic larvae develop over 22–27 days, progressing through five distinct developmental stages (see Section 2.4) influenced by light-sensitive and phototactic behavior with thermal tolerance [41,50,55,60,61]. This pattern is consistent with experimental evidence in other tropical holothurians. Ref. [55] evaluated the negative phototaxis and photokinesis of actively swimming auricularia larvae of *A. japonicus* under red light at 500 lx, meaning that they preferentially move away from the light source. This indicates a clear behavioral response, with larvae seeking areas farther from the surface to optimally position themselves in the water column. This should also apply to the auricularia larvae of *I. fuscus*, which exhibit considerable dispersal capacity across the eastern tropical Pacific (ETP) populations, ranging from Baja California, México, to northern Perú, including oceanic archipelagos, such as the Galápagos islands [62,63]; thus, it is capable of tolerating the thermal gradients and light fluctuations characteristic of the ETP, thereby enhancing their survival potential [64,65].

The data in this study align with the proposed conceptual model and are in accordance with other studies reporting temperature-mediated adaptive physiological plasticity in *I. fuscus* auricularia larvae, where thermal conditions critically regulate its growth performance and disease resistance [26,27,38,39,42,43]. Nevertheless, it is important to acknowledge that larval yield in hatcheries is driven by the interaction of several environmental and culture-related factors. For example, the adequate tank design and characteristics—such as, volume, shape, and color—played a critical role during the five larval development stages, promoting efficient swimming and stable positioning within the water column. This prevented larval surface aggregation and allowed them to occupy areas with favorable dissolved oxygen, temperature, and light conditions. All these conditions supported better water quality and reduced physiological stress, contributing to higher larval survival [19,66,67].

In contrast, the combined influence of temperature and light conditions, including larval rearing in black-colored tanks under reduced indoor light may trigger endocrine responses mediated by melatonin, commonly referred to as the “hormone of darkness”, associated with circadian regulation. According to the thermal mismatch hypothesis, this planktotrophic larva may obtain a physiological advantage—a form of thermal refuge—when environmental temperatures exceed the tolerance limits of the parasite while remaining within the viable range for the host. This pattern suggests that the auricularia larva possesses a distinct thermal performance curve, with optimal functioning across a particular temperature window. Furthermore, light enhanced larval physiological responsiveness. This condition may have contributed to improved immune regulation and reduced susceptibility to protozoan parasites at elevated temperatures (27 °C) [66]. Meanwhile, low-temperature conditions reduce larval metabolism (as hypometabolism with reduction in aerobic scope), which in turn drives physiological responses leading to dormancy or aestivation [41]. This explains the high prevalence and intensity of parasite infections, as well as the very low survival rates (<0.3%), where suboptimal rearing temperatures for *I. fuscus* larvae are those below 23 °C, particularly when pathogens are present.

**Figure 6:**
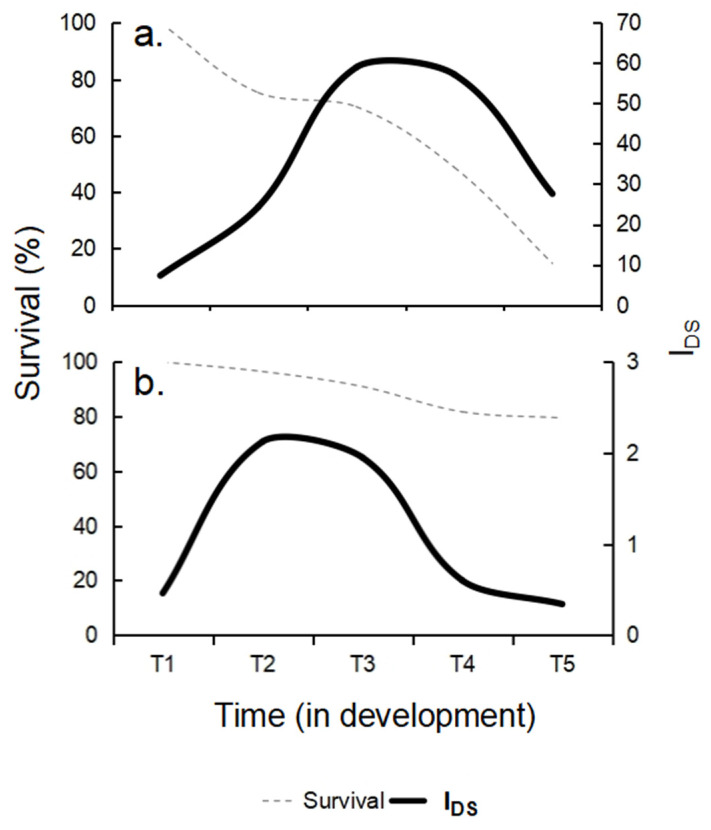
Relationship between survival and I_DS_ in auricularia of the sea cucumber *Isostichopus fuscus* with (LWP) and without parasites (LWOP) at five different phases of development and under two temperature regimes: (**a**) low temperature and (**b**) high temperature.

Elevated temperatures (see Figure 5 and Figure 6) may enable the host to partially escape or mitigate the effects of parasitic infections, as the parasites appear to be more thermally sensitive than the host. Under these conditions, relatively high temperature combined with moderate light levels may contribute to maintaining stable protozoan parasite loads without increasing host susceptibility, thereby reducing the risk of severe parasite-induced damage in the larvae. Importantly, such conditions are unlikely to impose additional metabolic costs that could compromise the maintenance of larval innate immune defenses [51,52,53]. When the temperature factor was experimentally isolated across the five auricularia larval stages, low-temperature conditions were associated with an increased progression of parasitic disease, whereas higher temperatures promoted improved larval performance and health. This response likely reflects temperature-dependent metabolic processes that regulate energy allocation and physiological resilience. Under high-temperature conditions, larvae developed more rapidly, completing the development in approximately 18 days while maintaining low parasite loads and exhibiting fewer disease symptoms. Active feeding and abundant fecal production further suggest efficient food assimilation and elevated metabolic activity under these conditions.

### 4.2. Phycosphere of Auricularia

Microalgae constitute the primary trophic foundation for the long-lived planktotrophic larvae of tropical sea cucumbers [16,21,26,67]. Therefore, the selection of nutritionally rich diets is a critical factor in hatchery production [68]. In particular, diets that provide a broad spectrum of essential nutrients—including proteins, carbohydrates, vitamins, hormones, and bioactive compounds—as well as high levels of highly unsaturated fatty acids (HUFAs), are essential for supporting the optimal larval development [69,70,71]. Therefore, mixed live microalgal assemblages are generally considered more nutritionally balanced than single-species diets and have been widely adopted in hatchery systems. Such multi-species microalgal diets represent a key determinant of larval performance, contributing to improved survival, growth, physiological condition, and overall production efficiency in *I. fuscus* [38,72].

In this study, *Chaetoceros gracilis*, *T-isochrisis lutea*, and *Rhodomonas* sp were supplied in a ratio of 4:1:1 at a final concentration from 1 × 10^4^ to 4 × 10^4^ cells·mL^−1^. *C. gracilis* (2.8–3.0 µm in size) has high protein (20–25%) and lipid (39%) content, with PUFA comprising 5–62% of total fatty acids [73]. *T. lutea* (3 × 5 µm in size) has high protein (40–49%) and lipid (20–30%) content [73,74,75], and *Rhodomonas* sp (10 × 12 µm in size) has high protein (42–59%) and lipid (12–29%) content [73,76]. *Rhodomonas* sp presents a high percentage of total PUFAs, EPAs, and DHAs (PUFAs = 64.35%, EPAs = 8.4% and DHAs = 6.9%), followed by *T. lutea* and *C. gracilis* [73,76]. It is necessary to highlight the importance of PUFAs as essential nutrients for auricularia larvae of *I. fuscus* in aquaculture [72], which might be modulated by the supplied diet. Refs. [70,76] suggested that larvae fed with *Rhodomonas* sp produce the highest lipid content, particularly PUFA (22:6n-3 and 20:5n-3), and high levels of carbohydrates, which reflects robust physiological condition and the capacity for successful metamorphosis [76]. Nonetheless, the microalgal assemblage used in this study may also influence the composition of associated bacterial communities adapted to auricularia larvae, including taxa such as *Vibrio* [56]. These microbial assemblages can perform a variety of ecological functions, including microbial signaling and other host–microbe interactions that shape the rearing environment. However, under certain conditions, they may also shift toward opportunistic pathogenic roles, potentially acting as vectors for disease through bacteria–protozoa consortium-like interactions. Such dynamics may influence larval health and survival in hatchery systems, particularly under low-temperature conditions that may favor pathogen persistence and proliferation.

Our data suggest that auricularia larvae with an I_DS_ under 40% (see Table 1) can transport lipid energy reserves from phytoplankton in the mature auricularia stage (particularly from the base of a nutrient-rich diet) to form up to 12 hyaline spheres—HSs [72]; however, the larvae with high parasite load did not form HSs (Figure 1, Figure 2 and Figure 3). HSs have a higher percentage of saturated fatty acids, dominated by the presence of myristic, palmitic, stearic, palmitoleic, and alfa-linolenic and EPA fatty acids. This content of fatty acids is decisive for completing this critical stage until the late auricularia stage. Ref. [77] successfully manipulated the size and number of HSs through alterations of the phytoplankton diet fed to auricularia. The occurrence of hyaline spheres (HSs) and associated granular structures appears to be an important feature during the larval development of *I. fuscus*. These structures may reflect physiological processes related not only to nutrient storage and metabolic regulation but also to the maintenance of an active innate immune system during early development. However, this pattern does not seem to be universal among holothurians. For example, successful metamorphosis has been reported in cultured larvae of *Apostichopus californicus* even in the absence of HSs, suggesting interspecific differences in developmental and physiological strategies. These observations indicate that the functional role of HSs in *I. fuscus* may extend beyond simple energy reserves and could be associated with maintaining larval physiological condition and immune competence during metamorphosis. Consequently, further research is required to determine whether HSs represent a physiological prerequisite for successful metamorphosis in healthy *I. fuscus* larvae and to clarify their role in the nutritional–immunological regulation of early larval development under hatchery conditions.

Consistent with the findings of [56], recent studies in tropical sea cucumber larvae demonstrate that diet quality not only regulates growth and survival but also shapes the associated microbiome. Therefore, diet does not just feed the larva, but it also “selects” its microbiome, and that modulates disease. For example, mixed diets have been shown to enhance microbial diversity and larval performance in *Holothuria leucospilota*, suggesting that nutritional conditions directly influence host’s physiological status through microbiome-mediated processes [78]. This supports the view that larval condition represents a central axis linking metabolism, microbial regulation, and susceptibility to disease in holothurian aquaculture systems.

In contrast, relatively low food rations (<5 × 10^4^ cells mL^−1^), when composed of nutritionally rich mixed microalgae, may contribute to reducing both the risk and severity of infectious diseases. Higher food availability can accelerate host growth and stimulate cellular metabolism, which may inadvertently favor pathogen proliferation and increase disease susceptibility. This mechanism may be particularly relevant for *I. fuscus* auricularia larvae, whose active swimming behavior as heterotrophic organisms may facilitate the motility and transmission of associated protozoan parasites [56,78,79,80]. Conversely, maintaining low but continuous food availability under near ad libitum conditions may improve the physiological status of the host while simultaneously limiting pathogen development. Under restricted feeding regimes, the reduced availability of resources can constrain pathogen growth and metabolic activity, given that parasite proliferation depends on the metabolic resources of the host [81]. For this reason, food levels in the present trial were carefully regulated to minimize resource competition and potential pathogen amplification.

### 4.3. Holobiome and Diseases in Auricularia

The auricularia larva of *I. fuscus* (as host) has a digestive system—mouth, esophagus, cardiac sphincter, stomach, intestine, and anus—that act as a dynamic core holobiome, with long-term effects on the host’s immunological and physiological development, and its disturbance is known to trigger various diseases in the host [42,44,45,82]. Although this study did not perform any bacterial analysis, we presume that the diseases observed in the larva are caused by bacteria-protozoa consortium-*like* interactions [44,45].

Increasing evidence indicates that the microbiome composition in holothurian larvae undergoes pronounced shifts throughout early ontogeny. For instance, Ref. [45] documented significant changes in the bacterial community structure across developmental stages (egg, auricularia, and doliolaria), revealing a stage-specific trajectory of microbial succession. Such dynamics may have important consequences for larval health, as alterations in the microbiome can facilitate opportunistic pathogenic interactions under stressful environmental conditions (e.g., temperature).

Temperature is widely recognized as a key regulator of host–parasite interactions in aquatic systems [17,28,83]. Several studies have shown that the infectivity and virulence of microparasites can vary markedly across thermal ranges, with important consequences for host health. For instance, thermal exposure experiments demonstrated that spores of the endoparasitic bacterium *Pasteuria ramosa* infecting *Daphnia* exhibit substantially reduced infectivity after exposure to elevated temperatures [84]. Similarly, parasite–host interactions can sometimes modulate host tolerance to environmental stressors; cestode larvae have been shown to increase resistance to pollutants and oxidative stress in *Artemia* even under moderately elevated temperatures [85]. Temperature-dependent parasite dynamics have also been documented in aquaculture species, such as Atlantic salmon (*Salmo salar*), where the infestations of the sea louse *Lepeophtheirus salmonis* were the highest at lower temperatures and declined markedly in warmer conditions [86]. Collectively, these findings support the view that parasite performance is often more thermally constrained than that of their hosts, suggesting that environmental temperature can strongly modulate disease dynamics. Consistent with this pattern, disease outbreaks in aquaculture systems, including shrimp farms in Ecuador, have frequently been associated with periods of unusually low seawater temperature [87,88]. 

In this context, larval diseases such as rotten edge syndrome—associated with *Vibrio lentus*—have been linked to thermal stress that increases host susceptibility to infection. Prolonged exposure to suboptimal low temperatures (<23 °C) can exacerbate this condition [27,41,89], likely by inducing metabolic imbalance and the accumulation of reactive metabolites during the onset of feeding in pelagic larvae [89,90,91]. Together, these findings highlight that larval microbiome assembly is jointly shaped by ontogenetic processes and culture conditions, and that disruptions in this balance may compromise larval health. Consequently, understanding host–microbiome–environment interactions is essential for improving larval resilience and ensuring sustainable sea cucumber aquaculture production systems [84]. In the present study, larval disease in *I. fuscus* was characterized by a set of concurrent clinical manifestations observed during early developmental stages [45,82,89,90]. These included: (i) stomach ulceration symptoms (SUSs), characterized by anorexia, an empty digestive tract, impaired motility, and erratic swimming behavior; (ii) rotting edge symptoms (RESs), marked by lethargy, thickening, and discoloration of the stomach wall toward a brownish tone, and delayed formation of larval structures; and (iii) stomach atrophy syndrome (SAS), evidenced by stomach shrinkage, collapse of the digestive structure, and high larval mortality (see Figure 3 and Figure 4). Notably, the earliest pathological signs and tissue lesions were predominantly detected within the first 3–7 days of development, particularly during the early auricularia stages (EA, MA-I, and MA-II), where affected larvae exhibited marked deviations from normal morphology and behavior. Importantly, these alterations appeared to be partially reversible under favorable temperature conditions, suggesting that thermal regimes may play a critical role in modulating disease progression and larval physiological resilience.

The highest parasite infestation occurred during the mature auricularia stage (T4), where heavily infected larvae frequently harbored more than 160 parasites per host. Under these conditions, the infected larvae exhibited reduced body length and a marked delay in organogenesis compared with parasite-free individuals, which developed more rapidly. This pattern suggests that high parasite burdens may impose substantial physiological costs on the host, likely diverting metabolic resources away from somatic growth and developmental processes. Moreover, clear temperature-dependent differences in developmental performance were observed. Mature auricularia larvae with low parasite loads (fewer than 10 parasites) or without detectable infection completed their development in approximately 18 days under high-temperature conditions, whereas heavily infected larvae maintained at lower temperatures required more than 25 days to reach comparable stages. Together, these findings suggest that parasite load and thermal conditions interact to strongly influence larval growth dynamics and developmental timing, highlighting the importance of environmental regulation in mitigating parasite-mediated impact on larval performance.

## 5. Conclusions

This study demonstrates that temperature is a key driver of host–parasite dynamics, metabolic performance, and developmental outcomes in auricularia larvae of the tropical sea cucumber *Isostichopus fuscus*. Elevated temperature (27 °C) enhanced larval metabolic activity, accelerated development, and reduced the severity of protozoan parasitic infections without compromising survival under culture conditions. Notably, mature auricularia (T4) with low or negligible parasite loads completed development within 18 days at high temperature, whereas heavily infected larvae reared under lower temperatures required more than 25 days. In contrast, prolonged exposure to suboptimal temperatures (<23 °C) induced severe pathological conditions in the digestive tract—manifested as SUSs, RES, and SAS—driven by true protozoan parasitism rather than endosymbiotic associations.

From a practical perspective, optimal larval rearing should combine a thermal regime of ~27 °C with controlled feeding strategies, maintaining stomach fullness at 70–85% using low-concentration mixed microalgal diets (1 × 10^4^–4 × 10^4^ cells mL^−1^) composed of *Chaetoceros gracilis*, *Tisochrysis lutea*, and *Rhodomonas* sp., the latter providing high levels of essential PUFAs (EPA and DHA).

These conditions collectively promote larval growth, physiological resilience, and reduced disease risk. Overall, these findings highlight the importance of environmental regulation in mitigating parasitism and optimizing larval performance, providing a robust framework for sustainable aquaculture of *I. fuscus*, particularly for the dark chocolate-brown morphotype. Further research should address the mechanistic links between environmental stress, host physiology, and parasite outbreaks across different broodstock morphotypes.

## Figures and Tables

**Table 1:** Metric of disease severity index (I_DS_) of physiological damage scale to the host, the auricularia larva of the sea cucumber *Isostichopus fuscus* [49].

Level	I_DS_ Range	Physiological Damage Scale	Description
I	1–20	Low	Minor effect.
II	20–40	Medium	Medium effect. The consequences are significant and can affect the achievement of larval culture.
III	40–60	High	High effect. The consequences can cause significant damage and mortality of larvae.
IV	60–80	Very high	Very high effect. The consequences are very serious and cause significant damage and massive disease.
V	80–100	Critical	Severe effect. The consequences are extremely serious and can lead to the failure of the project.

**Table 2:** The summary of the total number of parasites counted, the proportion of larvae with (+) and without (−), and the average of parasites for each time (or larval stage) and treatment in auricularia larva of the sea cucumber *Isostichopus fuscus* in the trial that lasted for 25 days.

Temperatures/Parameters	Times				
Low	T1	T2	T3	T4	T5
EA	100.0	11.3	3.7	0.0	0.0
MA 1	0.0	88.7	16.3	26.2	0.0
MA 2	0.0	0.0	80.0	2.5	0.0
MA	0.0	0.0	0.0	71.3	41.2
LA	0.0	0.0	0.0	0.0	58.8
N	80	71	67	59	47
Survival	100.0	88.8	83.8	73.8	58.8
Average total length ± ES	235.1 ± 2.0	520.9 ± 10.1	768.7 ± 16.3	1064.8 ± 11.7	630.9 ± 22.7
Prevalence	0.7	0.7	0.9	0.9	0.8
Intensity	11.1	33.6	71.4	60.7	39.5
DSI	7.0	24.0	59.0	57.0	28.0
**High**					
EA	100.0	0.0	0.0	0.0	0.0
MA 1	0.0	100.0	0.0	0.0	0.0
MA 2	0.0	0.0	100.0	0.0	0.0
MA	0.0	0.0	0.0	100.0	0.0
LA	0.0	0.0	0.0	0.0	100.0
N	80	76	72	72	63
Survival	100.0	95.0	90.0	90.0	78.8
Average total length ± ES	244.1 ± 2.7	558.1 ± 5.9	837.4 ± 9.0	1165.5 ± 11.8	594.2 ± 16.3
Prevalence	0.4	0.9	0.7	0.3	0.2
Intensity	1.3	2.5	2.2	2.3	6.3
DSI	0.5	2.1	2.0	0.6	0.3

**Table 3:** The summary of repeated measures analysis of variance with effect of temperature for infection parameters (prevalence, mean intensity, disease severity index, and survival) and parasite effect on the auricularia larvae of the sea cucumber *Isostichopus fuscus*.

Effect Sizes	SS	df	MS	F	*p*
Infection Parameters					
Prevalence					
Temperature	1.01336	1	1.01336	10.3180	0.018317
Error	0.58928	6	0.09821		
Time	0.57322	4	0.14331	3.3576	0.025619
Time × temperature	0.97844	4	0.24461	5.7312	0.002199
Error	1.02433	24	0.04268		
Mean Intensity					
Temperature	16,282.79	1	16,282.79	104.4381	0.000051
Error	935.45	6	155.91		
Time	4552.13	4	1138.03	3.9644	0.013118
Time × temperature	4453.48	4	1113.37	3.8785	0.014396
Error	6889.43	24	287.06		
Disease Severity Index					
Temperature	11,544.14	1	11,544.14	60.81290	0.000234
Error	138.9	6	189.83		
Time	4098.90	4	1024.73	5.00025	0.004476
Time × temperature	3924.85	4	981.21	4.78793	0.005542
Error	4918.44	24	204.93		
Survival					
Temperature	14,493.2	1	14,493.2	773.73	0.0000
Error	112.4	6	18.7		
Time	12,337.6	4	3084.4	240.94	0.0000
Time × temperature	3768.8	4	942.2	73.60	0.0000
Error	307.2	24	12.8		
Larval Size					
Total Length					
Parasites	17,731	1	17,731	17.90	0.001165
Temperature	111,353	1	11,353	11.46	0.005410
Parasites × temperature	5	1	5	0.00	0.947294
Error	11,884	12	990		
Time	6,770,525	4	12,631	1565.75	0.000000
Time × parasites	306,829	4	76,707	70.96	0.000000
Time × temperature	69,503	4	17,376	16.07	0.000000
Time × parasites × temperature	6785	4	1696	1.57	0.197752
Error	51,890	48	1081		

## Data Availability

The original contributions presented in this study are included in the article. Further inquiries can be directed to the corresponding author.
